# Deferasirox drives ROS-mediated differentiation and induces interferon-stimulated gene expression in human healthy haematopoietic stem/progenitor cells and in leukemia cells

**DOI:** 10.1186/s13287-019-1293-y

**Published:** 2019-06-13

**Authors:** Tiziana Tataranni, Carmela Mazzoccoli, Francesca Agriesti, Luciana De Luca, Ilaria Laurenzana, Vittorio Simeon, Vitalba Ruggieri, Consiglia Pacelli, Gerardo Della Sala, Pellegrino Musto, Nazzareno Capitanio, Claudia Piccoli

**Affiliations:** 1 Laboratory of Pre-Clinical and Translational Research, IRCCS-CROB, Referral Cancer Center of Basilicata, Rionero in Vulture, PZ Italy; 2 Department of Public, Clinical and Preventive Medicine, Medical Statistics Unit, University of Campania Luigi Vanvitelli, Caserta, Italy; 3 Department of Clinical and Experimental Medicine, University of Foggia, Foggia, Italy; 4 Hematology Department of Basilicata, IRCCS-CROB Referral Cancer Center of Basilicata, Rionero in Vulture, Italy

**Keywords:** DFX, ROS, Interferon signalling, Differentiation

## Abstract

**Background:**

Administration of the iron chelator deferasirox (DFX) in transfusion-dependent patients occasionally results in haematopoiesis recovery by a mechanism remaining elusive. This study aimed to investigate at a molecular level a general mechanism underlying DFX beneficial effects on haematopoiesis, both in healthy and pathological conditions.

**Methods:**

Human healthy haematopoietic stem/progenitor cells (HS/PCs) and three leukemia cell lines were treated with DFX. *N*-Acetyl cysteine (NAC) and fludarabine were added as antioxidant and STAT1 inhibitor, respectively. In vitro colony-forming assays were assessed both in healthy and in leukemia cells. Intracellular and mitochondrial reactive oxygen species (ROS) as well as mitochondrial content were assessed by cytofluorimetric and confocal microscopy analysis; mtDNA was assessed by qRT-PCR. Differentiation markers were monitored by cytofluorimetric analysis. Gene expression analysis (GEA) was performed on healthy HS/PCs, and differently expressed genes were validated in healthy and leukemia cells by qRT-PCR. STAT1 expression and phosphorylation were assessed by Western blotting. Data were compared by an unpaired Student *t* test or one-way ANOVA.

**Results:**

DFX, at clinically relevant concentrations, increased the clonogenic capacity of healthy human CD34^+^ HS/PCs to form erythroid colonies. Extension of this analysis to human-derived leukemia cell lines *Kasumi-1*, *K562* and *HL60* confirmed DFX capacity to upregulate the expression of specific markers of haematopoietic commitment. Notably, the abovementioned DFX-induced effects are all prevented by the antioxidant NAC and accompanied with overproduction of mitochondria-generated reactive oxygen species (ROS) and increase of mitochondrial content and mtDNA copy number. GEA unveiled upregulation of genes linked to interferon (IFN) signalling and tracked back to hyper-phosphorylation of *STAT1*. Treatment of leukemic cell lines with NAC prevented the DFX-mediated phosphorylation of STAT1 as well as the expression of the IFN-stimulated genes. However, STAT1 inhibition by fludarabine was not sufficient to affect differentiation processes in leukemic cell lines.

**Conclusions:**

These findings suggest a significant involvement of redox signalling as a major regulator of multiple DFX-orchestrated events promoting differentiation in healthy and tumour cells. The understanding of molecular mechanisms underlying the haematological response by DFX would enable to predict patient’s ability to respond to the drug, to extend treatment to other patients or to anticipate the treatment, regardless of the iron overload.

**Electronic supplementary material:**

The online version of this article (10.1186/s13287-019-1293-y) contains supplementary material, which is available to authorized users.

## Background

Deferasirox (DFX) is a once-daily oral iron chelator primarily used to reduce chronic iron overload in patients receiving blood transfusions for various chronic anaemias [1] such as myelodysplastic syndrome (MDS) [2]. Interestingly, a series of case reports of transfusion-dependent MDS patients treated with DFX [3–5], now supported by systematic clinical trials [6–12], show an increase in hemoglobin, platelets and neutrophils in a variable subset of patients with an achievement of transfusion independence as well as an improvement of haematopoiesis. Recent studies demonstrated the efficacy of DFX at not conventional low doses [13], suggested its feasible utilization also in other patients’ groups for whom iron chelating therapy is generally not recommended [14] and showed a significant beneficial effect on haematopoietic recovery in patients submitted to allogeneic haematopoietic stem cell transplantation [15]. These findings would suggest the possibility to extend patient categories eligible for DFX treatment as well as to revise the optimal dose to achieve haematological improvement, independently on its iron chelating activity. However, despite the increasing number of clinical data, the mechanism of action of this peculiar property of DFX is still under investigation. Our group had already shown a possible interplay between DFX-mediated reactive oxygen species (ROS) production, activation of regulatory factors, mitochondrial biogenesis and erythroid commitment in healthy haematopoietic stem/progenitor cells (HS/PCs) [16]. In this setting, ROS would act as secondary messengers, able to affect several physiological processes [17] and it is conceivable they can act as mediators of stem cell processes, including self-renewal and differentiation [18, 19]. DFX ability to recover disorders of haematopoietic cell differentiation suggests it could also act on pathways and modulators of cell fate, altered in several pathologic conditions. The present study aims to deepen our previously obtained data, extending our analysis also to other cellular models of haematological diseases, in the attempt to elucidate a general molecular mechanism underlying DFX beneficial effect on haematopoiesis.

## Methods

### Reagents

Deferasirox (DFX) from (Novartis, Basel, Switzerland), deferoxamine (DFO), *N*-acetyl cysteine (NAC) and fludarabine from Sigma Aldrich, St. Louis, MO, were freshly diluted to required concentrations with culture medium before use.

### Human samples

HS/PCs (haematopoietic stem/progenitor cells) mobilized from adult healthy subjects’ human bone marrow (hBM) were obtained from healthy donors by apheresis performed for allogeneic transplant purpose, after mobilization following recombinant granulocyte colony-stimulating factor treatment. HS/PCs were collected via a code/spectra device and positively selected by the super magnetic iron-dextran particles directly conjugated to anti-CD34 monoclonal antibodies. All donors gave informed consent according to the Declaration of Helsinki. Human umbilical cord blood (hUCB) was obtained as previously described [20]. This study was performed according to the Regional Institutional Review Board (IRB) Statement (no. 20140040750; date: 11/18/2014).

### Cell lines

The human cell lines Kasumi-1, K562 and HL60 were acquired from American Type Culture Collection (Rockville, MD, USA). All cell lines were grown at 37 °C in 5% CO_2_ in culture medium supplemented with 1% of penicillin–streptomycin (Gibco) and 2 mM of l-glutamine (Gibco); Kasumi-1 cells were maintained in RPMI-1640 medium (Gibco) supplemented with 20% FBS, K562 cells in RPMI-1640 medium with 10% FBS and HL60 cells in IMDM medium (Gibco) with 20% FBS.

### Colony assay

In vitro colony-forming assays were carried out from mobilized HS/PCs of human adult healthy donors (hmBM) or human umbilical cord blood (hUCB) and from Kasumi-1, K562 and HL60 cells, according to the manufacturer protocol. Each cell type was resuspended in IMDM medium and mixed with methylcellulose-based cultures (MethoCult-H4434; StemCell Technologies, Inc., Vancouver, BC, Canada), with or without DFX 20 μM. Cultures were then incubated at 37 °C in a fully humidified atmosphere supplemented with 5% CO_2_. Two weeks later, the growth of colony-forming unit-granulocyte/macrophage (CFU-GM) colonies, burst-forming unit-erythroid (BFU-E) colonies and colony-forming unit-granulocyte, erythroid, macrophage megakaryocytes (CFU-GEMM) colonies was scored under an inverted microscope (magnification × 40), and the colonies formed were enumerated. Each assay was performed in duplicate. Per each source of cells, three independent experiments were performed.

### Cytofluorimetric assessment of intracellular and mitochondrial reactive oxygen species (ROS) and mitochondrial mass

Intracellular and mitochondrial ROS, as well as mitochondrial mass, were measured using flow cytometric analysis. 2 × 10^5^ cells per condition were incubated for 15 min at 37 °C with 2 μM of 2′,7′-dichlorofluorescein (DCF) (Sigma Aldrich, St. Louis, MO) to detect intracellular ROS, 5 μM MitoSOX™ (Molecular Probes, Eugene, OR, USA) to label superoxide, specifically produced by mitochondria, and 100 nM MitoTracker Green (Molecular Probes, Invitrogen Corp., Carlsbad, CA, USA), a labeling mitochondria probe. After washing in PBS, 10^4^ events for each sample were acquired using a Navios flow cytometer and analysed with Kaluza Analysis 1.3 software.

### Cytofluorimetric analysis of cell surface markers

Approximately 2 × 10^5^ cells per condition were incubated in the dark at room temperature for 15 min with the following directly conjugated monoclonal antibodies (MoAbs; BDB, San Jose, CA): CD14-APC and CD36-FITC for Kasumi-1 cells; CD71-FITC and CD36-FITC for K562; CD11b-PE and CD16-APC for HL-60. Cytofluorimetric analysis was performed using a Navios flow cytometer and Kaluza Analysis 1.3 software.

### Live cell imaging of mitochondrial ROS and mitochondrial mass

After 24 h treatment, 2 × 10^5^ cells were seeded on Cell Tak™-coated 35-mm glass bottom dishes. Following adhesion, cells were incubated for 15 min at 37 °C with 2.5 μM Mitosox or 0.5 μM Mitotracker-red. Stained cells were washed with PBS, and the fluorescent signals emitted by probes were examined with a Leica TCS SP8 confocal laser scanning microscope. Acquisition, storage and data analysis were performed with dedicated instrumental software from Leica.

### Gene expression profile

Total RNA was extracted by Trizol reagent (Life Technologies, Paisley, UK), according to the manufacturer’s instruction from HS/PCs treated or not with DFX or DFO 100 μM for 24 h (two samples per group); RNA concentration was determined on a Nanodrop spectrophotometer (Nano-Drop, Wilmington, DE) and quality assessed with the Agilent RNA 6000 Nano Kit on an Agilent 2100 Bioanalyzer (Agilent Technologies, Milan, Italy). For each sample, 300 ng of total RNA was reverse transcribed to synthesize cDNA and biotinylated cRNA according to the Illumina TotalPrep RNA amplification protocol (Ambion; category n. IL1791). Hybridization of 750 ng of cRNA on Illumina HumanHT12 v4.0 Expression BeadChip array (Illumina Inc.), staining and scanning were performed according to the standard protocol (Illumina Inc.). BeadChip was dried and scanned with an Illumina HiScanSQ system (Illumina Inc.). The intensity files were loaded into the Illumina Genome Studio software for quality control and gene expression analysis. Quantile normalization algorithm was applied on the data set to correct systematic errors: values below a detection score of 0.05 were filtered out and missing values were imputed. Microarray data (raw and normalized) were submitted to Array Express (accession number E-MTAB-6658). DFX- or DFO-treated cells were compared to untreated HS/PCs. Differently expressed genes (DEGs) were selected with differential score (DiffScore) cutoff set at ± 13 (*p* < 0.05) and logFC of ± 0.3. For DFX, the DEG list included 188 genes (109 upregulated and 79 downregulated) (Additional file 5: Table S1), and for DFO, the DEG list included 48 genes (34 upregulated and 14 downregulated) (Additional file 6: Table S2). DEG lists were used to evaluate the functional behavior in terms of biological processes performing an enrichment analysis with Ingenuity Pathway Analysis (IPA) (Ingenuity Systems, Redwood City, CA; https://www.qiagenbioinformatics.com/products/ingenuity-pathway-analysis/).

### Reverse transcription and real-time PCR analysis

One microgram of total RNA, isolated and quantified as described in the gene expression profile paragraph, was used in a reverse transcription (RT) reaction using the Transcriptor first strand cDNA synthesis kit (Roche Diagnostic, Penzberg, Germany) according to the manufacturer’s instructions. Quantitative Real-Time polymerase chain reaction (PCR) was performed in duplicate, using the QuantiTect Primer Assay (Qiagen, Basel, Switzerland) (Table 1). For each reaction, 2 μl of the complementary DNA (cDNA) was added to 16 μl of Light Cycler® 480SYBR Green I Master) (Roche) and primer pair, in a total volume of 20 μl. Quantification of the mRNA levels was performed on a LightCycler® 480 Real-Time PCR Instrument. The relative amounts of target genes were normalized with a stable housekeeping gene such as GAPDH by Light Cycler® 480 Software version 1.5 (ROCHE) using the 2ΔΔCt method.

**Table 1:** Features of the primers used for real-time PCR

Transcript	*T*_ann_ (°C)	Length (bp)	NCBI Ref Seq
ISG15	60	77	NM_005101
IFIT1	60	102	NM_001001887
IFITM1	60	94	NM_003641
IFITM2	60	88	NM_006435
IFITM3	60	85	NM_021034
OAS1	60	101	NM_002534
OAS3	60	90	NM_006187
EIF2AK2	60	93	NM_001135652
IRF7	60	111	NM_004030

### Mitochondrial DNA quantification

The measurement of mtDNA copy number, relative to nuclear DNA copy number was determined as previously described [21].

### Western blotting

Aliquots of 40 μg of proteins from each lysate were subjected to SDS polyacrylamide gel electrophoresis and transferred to a polyvinylidene difluoride membrane (Bio-Rad Laboratories; Hercules, CA, USA) by Trans Blot Turbo Transfer System. Membranes were probed with the primary antibody pSTAT1^(Tyr701)^ (rabbit anti-pSTAT1 ^Tyr701^ 1:2000 Cell Signaling Technology) and then incubated with anti-rabbit secondary antibody horseradish peroxidase-conjugated. The intensity of bands was normalized to total STAT expression (rabbit-antiSTAT1 1:1000 Cell Signaling Technology) and to β-ACTIN (15,000, SIGMA Aldrich, St. Louis, MO, USA). Signals were developed using the enhanced chemiluminescence kit (ClarityTM Western ECL Substrate, Bio-Rad) by ChemiDoc Imaging System XRS+ (BioRad) and analysed with the Image Lab 4.1 software.

### Statistical analysis

Experimental data were shown as mean ± standard deviation (SD) or mean ± standard error of the mean (SEM). Data were compared by an unpaired Student *t* test or one-way ANOVA followed by post hoc comparisons. *p* value less than 0.05 was considered as statistical significance. All analyses were performed using GraphPad Prism (GraphPad software, San Diego, CA, USA).

## Results

### DFX increases the capacity of healthy HS/PCs to form erythroid colonies

**Fig. 1:**
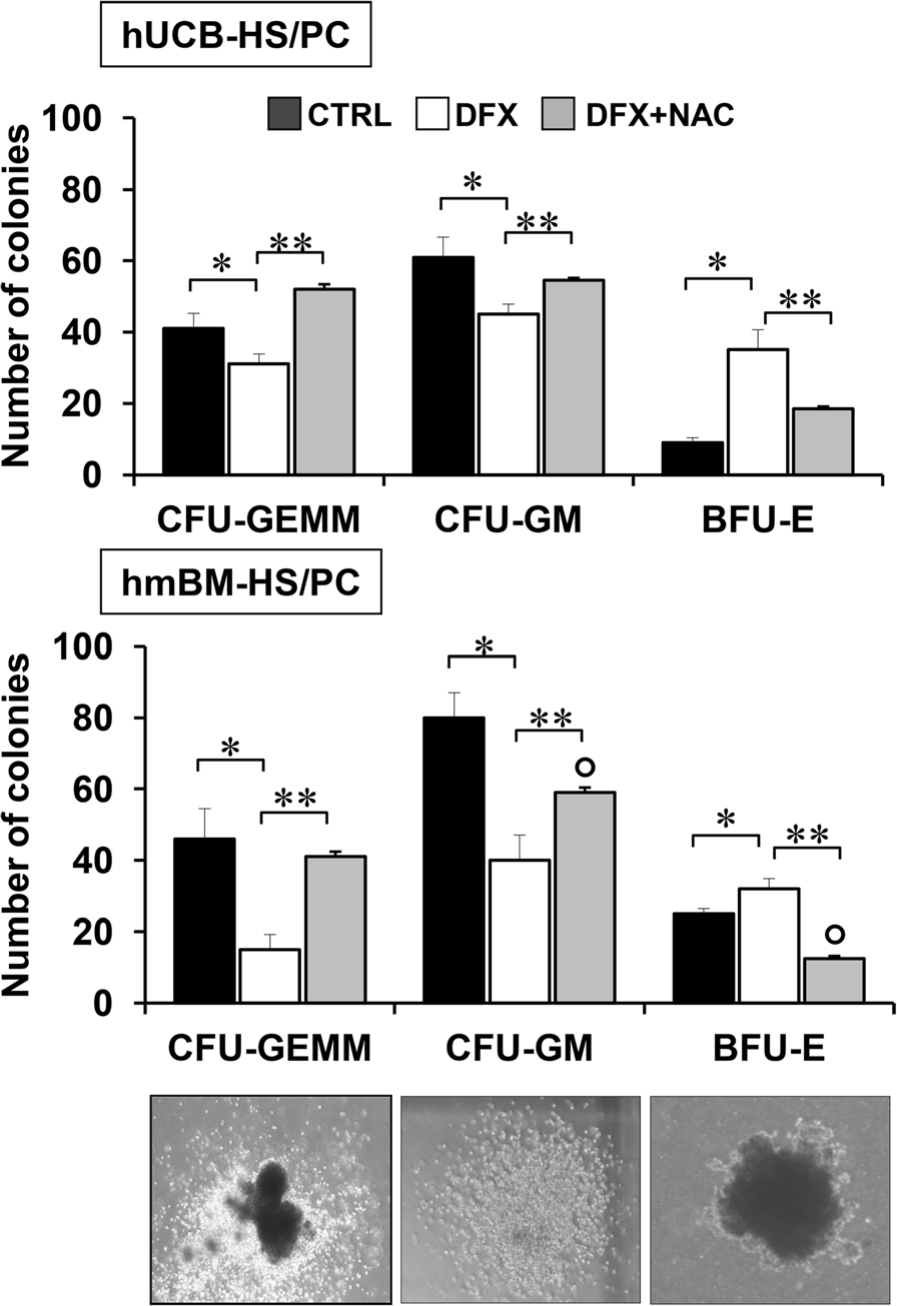
DFX enhances BFU-E colony formation proficiency of CD34^+^ HS/PCs. Quantitative analysis of CFU-GEMM, CFU-GM and BFU-E derived by CD34^+^ cells isolated from human umbilical cord blood (hUCB; upper panel) or mobilized bone marrow-derived from adult healthy subjects (hmBM; lower panel) and cultured without DFX, with 20 μM DFX and with 20 μM DFX + NAC 10 mM. DFX and NAC were added to methylcellulose-based medium at time of plating, and colonies were detected and enumerated after 14 days of culture. Results shown represent the mean ± SD of three independent experiments. (*p < 0.05 vs CTRL). Representative images of CFU-GEMM, CFU-GM and BFU-E scored under an inverted microscope (magnification × 40) are shown

In a previous study, we showed the proneness of circulating CD34^+^ HS/PCs to differentiate towards erythroid lineage following 100 μM DFX treatment. To further validate that observation, we performed in vitro clonogenic assays from human CD34^+^ HS/PCs either mobilized from bone marrow (hmBM) or isolated from umbilical cord (hUCB). For the long-time treatment requirement of 14 days, we added DFX in the methylcellulose base cultures at a final concentration of 20 μM, a safe effective dose for drug chronic exposure. As shown in Fig. 1, DFX treatment caused both in hmBM- and hUCB-derived CD34^+^ HS/PC a significant increase in the number of BFU-E (erythrocyte burst-forming units) colonies, more pronounced in hUCB-CD34^+^ cells but statistically significant also in hmBM-CD34^+^ HS/PCs. Conversely, both granulo-macrophage colonies (CFU-GM) and granulocyte, erythroid, macrophage, megakaryocytic colony-forming units (CFU-GEMM) decreased in DFX-treated HS/PCs regardless of the cell source. Notably, co-incubation of DFX with 10 mM of the antioxidant *N*-acetyl cysteine (NAC) prevented or mitigated the above-described effects. This result, based on a functional approach, strongly supports our previous data [16], thus providing further evidence that DFX conditioning of haematopoietic progenitor cells fosters their differentiation potential towards the erythroid lineage through ROS signalling activation.

### DFX induces ROS production and mitochondrial biogenesis in leukemia cell lines

**Fig. 2:**
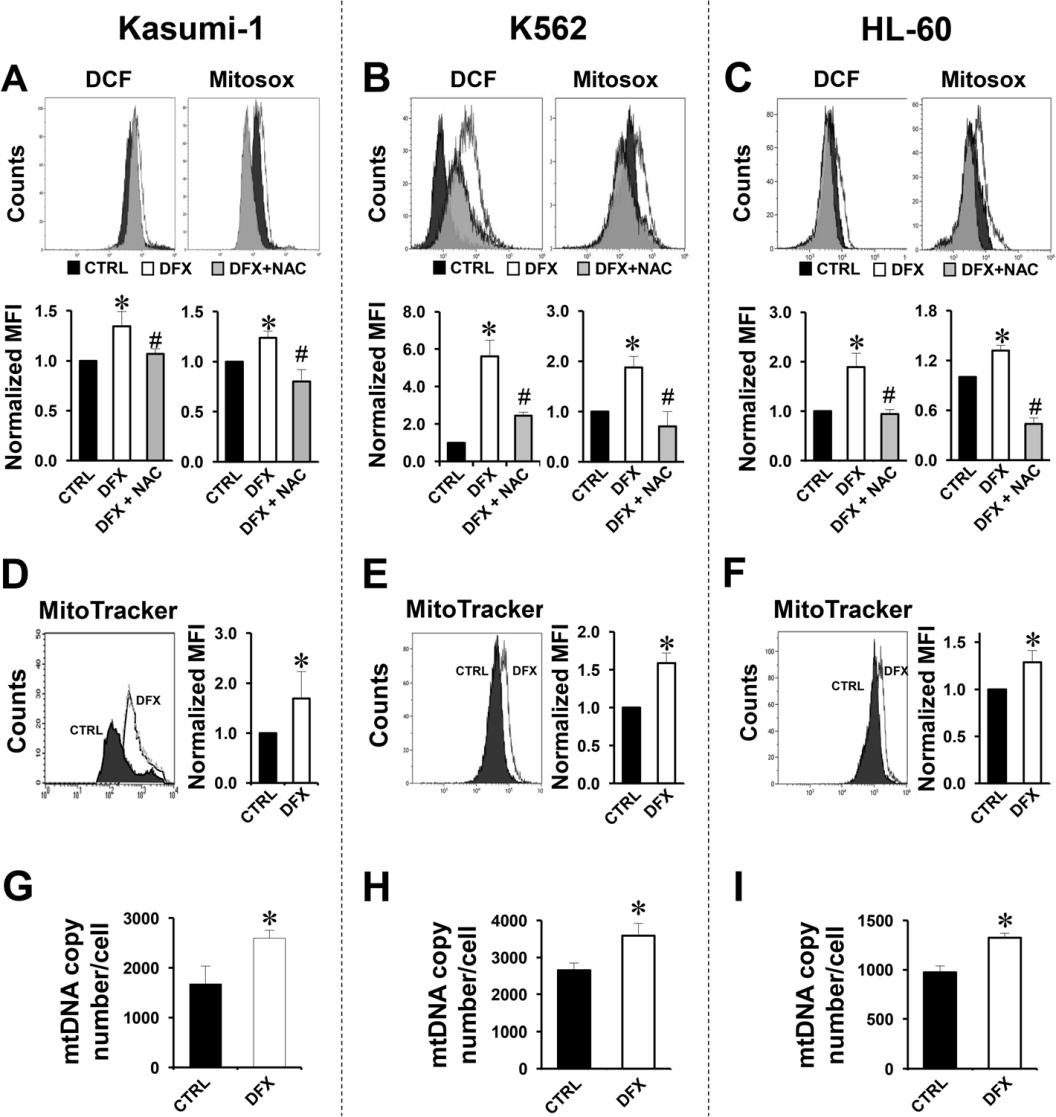
Effect of DFX on ROS production, mitochondrial mass and mtDNA in Kasumi-1, K562 and HL60. **a**–**c** Representative flow cytometric analysis of intracellular and mitochondrial ROS assessed by DCF (left panel) and Mitosox (right panel) probes in Kasumi-1 (**a**), K562 (**b**) and HL60 (**c**) treated with 100 μM DFX ± 10 mM NAC for 24 h. Bar histograms below each panel: normalized MFI of at least three experiments, expressed as mean ± SEM (*p < 0.05 vs CTRL, ^#^*p* < 0.05 vs DFX). **d**–**f** Representative flow cytometry analysis of Mitotracker green distribution in Kasumi-1 (**d**), K562 (**e**) and HL-60 (**f**). Per cell line, untreated cell distribution is represented with a black area, and 100 μM DFX-treated cells are shown as a white area. The panel on the right shows the quantitative analysis of normalized MFI as average ± SEM of three independent experiments (*p < 0.05 vs CTRL). **g**–**i** mtDNA copy number assessed by q-RT-PCR in Kasumi-1 cells (**g**), K562 (**h**) and HL-60 (**i**); the bar histogram shows values normalized to the nuclear DNA and are means ± SEM of three independent determination on biological replicates (*p < 0.05 vs CTRL)

In order to understand whether the DFX ability to alter the differentiation program was limited to healthy cells or extended to cellular models of human diseases, we decided to test the effect of DFX on three different leukemic cell lines, Kasumi-1, K562 and HL60. Firstly, we decided to assess if DFX interfered with the redox balance in the selected leukemic cell lines. To this aim, we used dichlorofluorescein (DCF) and MitoSox to probe intracellular ROS and mitochondrial superoxide anion (O_2_^•−^). As shown in Fig. 2a–c, the DCF-related fluorescence, detected by flow cytometry, was significantly enhanced following DFX treatment in all the three cell lines analysed. Likewise, also the MitoSox-related fluorescence resulted significantly increased in DFX-treated cells, indicating mitochondria as the major source of ROS. In both assays, the ROS-dependent fluorescence signal was significantly reduced by the co-incubation of DFX with the antioxidant (NAC) thus showing the specificity of the fluorescence signal observed. Confocal microscopy imaging performed by using the same probes confirmed in Kasumi-1 the ability of DFX to induce ROS production (Additional file 1: Figure S1A). Next, we detected the mitochondrial content using the specific probe MitoTracker Red. Figure 2d–f show that DFX treatment caused a significant 30–70% increase in mitochondrial mass in all the three leukemic cell lines as compared with untreated samples. The reported flow cytometry result was confirmed in Kasumi-1 by confocal microscopy imaging using the same probe (Additional file 1: Figure S1B). Accordingly, the relative mitochondrial DNA copy number was also increased after DFX treatment in all the three cell lines (Fig. 2g–i). All together, these results suggest a DFX-induced and, likely, redox signalling-mediated rewiring of the cell metabolism purposed to cope with the increased bioenergetic demand of proliferating/differentiating cells.

### Effect of DFX on differentiation in leukemia cells

**Fig. 3:**
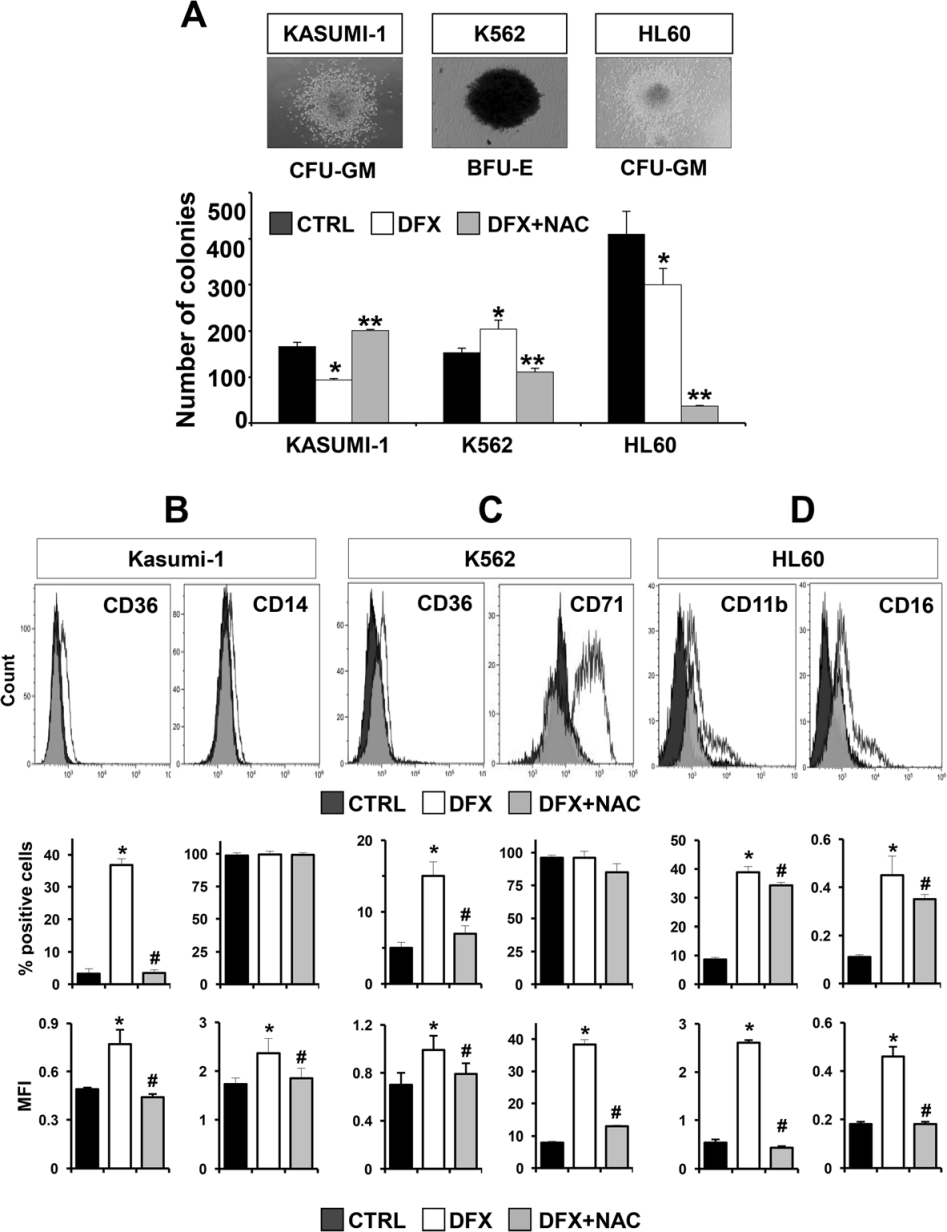
DFX affects colony formation ability and promotes expression of differentiation markers in leukemia cell lines. **a** Colony formation assay in Kasumi-1, K562 and HL60 treated with DFX and DFX + NAC. Representative images of colonies scored under an inverted microscope (magnification × 40) are shown on the top; left photo: CFU-GM from Kasumi-1 cells; central photo: BFU-E from K562 cells; right photo: CFU-GM from HL-60 cells. On the bottom, quantitative analysis of colonies derived by each cell line cultured without DFX, with 20 μM DFX and with 20 μM DFX + NAC 10 mM for 14 days in a methylcellulose-based medium. Colony number is shown as the mean ± SD of three independent experiments (*p < 0.05 vs CTRL). **b** Representative flow cytometric analysis of CD36 (on the left) and CD14 (on the right) distribution in untreated (CTRL—black area), DFX-treated (white area) and DFX + NAC (grey area) Kasumi-1 cells for 48 h. **c** Representative flow cytometric analysis of CD36 (on the left) and CD71 (on the right) distribution in untreated (CTRL—black area), DFX-treated (white area) and DFX + NAC (grey area) in K562 cells 48 h. **d** Representative flow cytometric analysis of CD11b (on the left) and CD16 (on the right) distribution in untreated (CTRL—black area), DFX-treated (white area) and DFX + NAC (grey area) in HL60 cells for 48 h. Per marker, histograms concerning both percentage of positive cells and MFI (expressed as mean ± SEM) are shown (*p < 0.05 vs CTRL, ^#^*p* < 0.05 vs DFX)

To directly test the ability of DFX to affect differentiation in leukemic cell lines, we first performed in vitro clonogenic assays. Figure 3a shows representative colonies derived from the Kasumi-1, K562 and HL-60 cell lines. Colony counting revealed a significant reduction of CFU-GM generated from Kasumi-1 and HL-60 after DFX treatment and an increased number of BFU-E from K562. Interestingly, NAC co-incubation counteracted the DFX action in Kasumi-1 and K562 colony assays, but not in the HL60 cell line where, instead, a dramatic impairment in the CFU-GM colony formation was observed. Our functional analysis was implemented by the evaluation at 24 h (Additional file 2: Figure S2) and 48 h of DFX treatment and NAC co-incubation on the expression of specific differentiation surface markers. In particular, in the Kasumi-1 cell line, we monitored and quantified the expression of CD36 and CD14, both upregulated during monocyte differentiation (Fig. 3b); in the K562 cell line, the expression of CD36 and CD71, markers of erythroid differentiation (Fig. 3c); and in the HL60 cell line, the expression of CD11b and CD16, typically upregulated at different stages *during* granulocyte differentiation (Fig. 3d). In cases where the percentage of positive cells was approximately 100% already at a basal level, we also reported the mean fluorescence intensity (MFI) of DFX ± NAC treatment. Cytofluorimetric analysis revealed that DFX was able to upregulate in a NAC-sensitive manner the expression of all the markers evaluated per cell line as inferred from the percentage of positive cells and/or the MFI.

### DFX activates interferon signalling pathway in healthy HS/PCs and leukemic cell lines

**Fig. 4:**
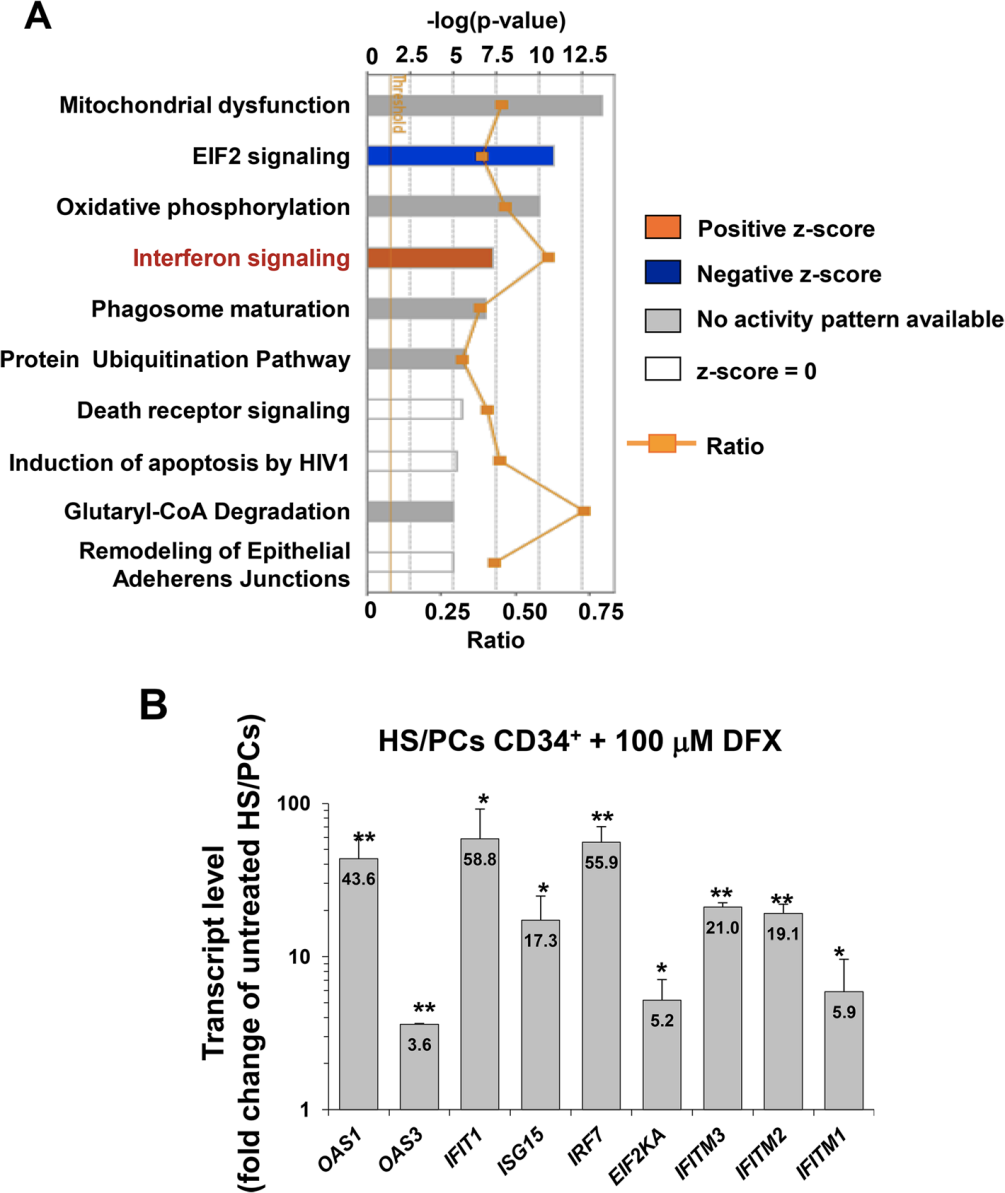
Ingenuity Pathway Analysis (IPA) and validation of differentially expressed genes in DFX-treated hmBM-HS/PCs. **a** Top 10 canonical pathways identified by IPA “Core Analysis” from genes changed more than 1.1-fold (*p* ≤ 0.05). Bars represent −log (*p* value) for significance; orange lines represent the ratio of changed genes to the total number of genes in the specific pathway. The IPA predicted one pathway having a positive *z*-score (predicted activation, orange bar), one pathway with a negative *z*-score (predicted inhibition, blue bar), 5 pathways having no activity/inhibition pattern predictable, in which the *z*-score could not be calculated. The *z*-score of zero corresponded to the standard mean of the normal distribution curve. **b** Real-time PCR of differentially expressed genes in CD34^+^ hmBM-HS/PCs treated with DFX. Quantitative reverse transcriptase polymerase chain reaction analysis is shown for transcripts of *OAS1*, *OAS3*, *IFIT1*, *ISG15*, *IRF7*, *EIF2KA*, *IFITM3*, *IFITM2* and *IFITM1* in HS/PCs treated with 100 μM DFX for 24 h. The values are the mean ± SD of normalized transcript levels of three independent experiments performed with different preparations of HS/PCs isolated from different donors. Per gene evaluated, fold change value of the transcript level in DFX-treated cells compared to that of untreated cells (CTRL) is reported inside the bar (*p < 0.05 vs CTRL; **p < 0.01 vs CTRL)

To clarify the mechanism by which DFX stimulated haematopoiesis, we performed a gene expression profiling in HS/PCs treated with 100 μM DFX for 24 h: 188 differently expressed genes were detected, particularly 109 upregulated and 79 downregulated in cells treated with DFX as compared to the control (*p* < 0.05) (Additional file 5: Table S1). Among the top ten canonical pathways derived from Ingenuity Pathway Analysis (IPA), interferon signalling was enriched in DFX dataset (Fig. 4a) with several differentially expressed genes resulting overexpressed as compared to untreated cells (Additional file 3: Figure S3 and Table 2).

**Table 2:** Selected list of IFN-induced genes

Symbol	ENTREZ_ID	ctrl_avgs	dfx_avgs	logFC	*p* value	Diff score
IFIT1	3434	309.7	4262.2	3.78	2.79E-02	15.5
ISG15	9636	1110.6	12,162.6	3.45	< 0.0001	338.4
IFITM1	8519	602.5	5394.2	3.16	< 0.0001	92.6
IFITM3	10,410	968.4	7529.1	2.96	< 0.0001	338.4
OAS3	4940	231	1645	2.83	< 0.0001	66.2
IRF7	3665	304	1612.9	2.41	< 0.0001	126.8
STAT1	6772	870.8	4038.8	2.21	1.19E−02	19.3
OAS1	4938	181.2	829	2.19	4.50E−04	33.4
EIF2AK2	5610	865.6	3166	1.87	< 0.0001	76.0
IFITM2	10,581	1547.2	5478	1.82	< 0.0001	111.7

Among these, we focused our attention on a group of upregulated genes to validate the gene expression analysis by quantitative RT-PCR. As shown in Fig. 4b, the transcript levels of *OAS1*, *OAS3*, *IFIT1*, *ISG15*, *IRF7*, *EIF2AK2*, *IFITM3*, *IFITM2* and *IFITM1* were significantly upregulated following DFX treatment, thus confirming that DFX positively affects the interferon signalling pathway. We also performed a similar analysis in HS/PCs treated with the iron-chelator deferoxamine (DFO) at the same doses and times of DFX. Among 48 differentially expressed genes detected (34 upregulated and 14 downregulated as compared to CTRL (*p* < 0.05)) (Additional file 6: Table S2), we did not find transcripts related to IFN signalling, as confirmed by the top ten canonical pathways derived from IPA (Additional file 4: Figure S4). This result would suggest specific effects elicited by DFX not shared with those of other iron chelating compounds.

**Fig. 5:**
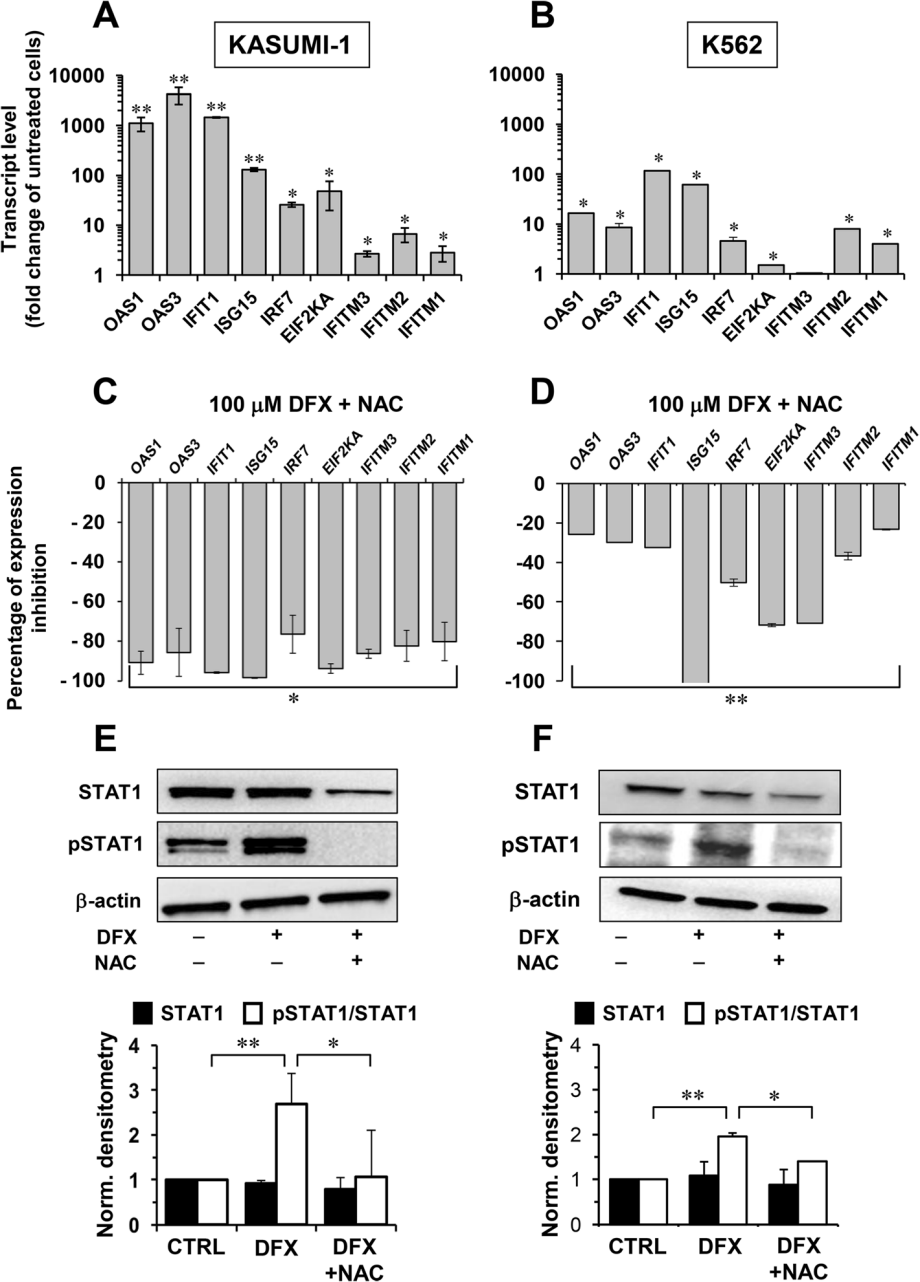
IFN gene expression and STAT1 phosphorylation, induced by DFX, are mediated by ROS. **a**, **b** Quantitative reverse transcriptase PCR analysis for *OAS1*, *OAS3*, *IFIT1*, *ISG15*, *IRF7*, *EIF2KA*, *IFITM3*, *IFITM2* and *IFITM1* in Kasumi-1 (**a**) and K562 (**b**) cells treated with 100 μM DFX for 24 h. The values are the mean ± SEM of normalized transcript levels of three independent experiments. Per gene evaluated, fold change value of the transcript level in DFX-treated cells compared to untreated cells (CTRL) is reported inside the bar (*p < 0.05 vs CTRL; **p < 0.01 vs CTRL). **c**, **d**: Percentage of expression-inhibition of IFN-related gene transcript levels in Kasumi-1 (**c**) and K562 (**d**) cells treated with 100 μM DFX + 10 mM NAC, compared to 100 μM DFX only treatment (*p < 0.05 vs DFX-only-treated cells; **p < 0.01 vs DFX-only-treated cells). **e**, **f** Western blot analysis of total STAT1 and pSTAT1^tyr701^ on whole protein Kasumi-1 (**e**) and K562 (**f**) cell extracts. Cells were treated with 100 μM DFX ± 10 mM NAC for 24 h. Per cell type, the panel on the top shows representative blots and bar histograms on the bottom represent densitometric analysis showing mean ± SEM of total STAT1 normalized to β-ACTIN (black bars) and p-STAT1/STAT1 ratio (white bars) of three independent experiments (*p < 0.05 vs CTRL; **p < 0.05 vs DFX)

The effect of DFX on the IFN signalling pathway was also examined in leukemia cells. Figure 5a, b show that treatment of Kasumi-1 or K562 cell lines with 100 μM DFX for 24 h recapitulated substantially the upregulation of the IFN-regulated genes observed in healthy HS/PCs. Next, we interrogated whether the altered redox tone elicited by DFX treatment was linked to the rewiring of the gene expression profile. Figure 5c, d show that NAC affected the DFX-mediated upregulation of the expression of all the selected IFN-regulated genes albeit the effect was stronger and broader in the Kasumi-1 than in the K562 cell line. A major transcription factor involved in the IFN signalling pathway is the signal transducer and activator of transcription 1 (STAT1) whose activation is positively regulated by phosphorylation. In keeping this notion, we assessed the content and phosphorylation state of STAT1 by Western blotting analysis using specific antibodies. Figure 5e, f show that DFX treatment of the Kasumi-1 and K562 cell lines caused a significant increase of the relative phosphorylation state of STAT1. Most notably, this effect was fully prevented by co-treatment with NAC.

### STAT1 and IFN-related signalling pathway are not involved in the DFX-mediated differentiation of Kasumi-1 and K562 cells

**Fig. 6:**
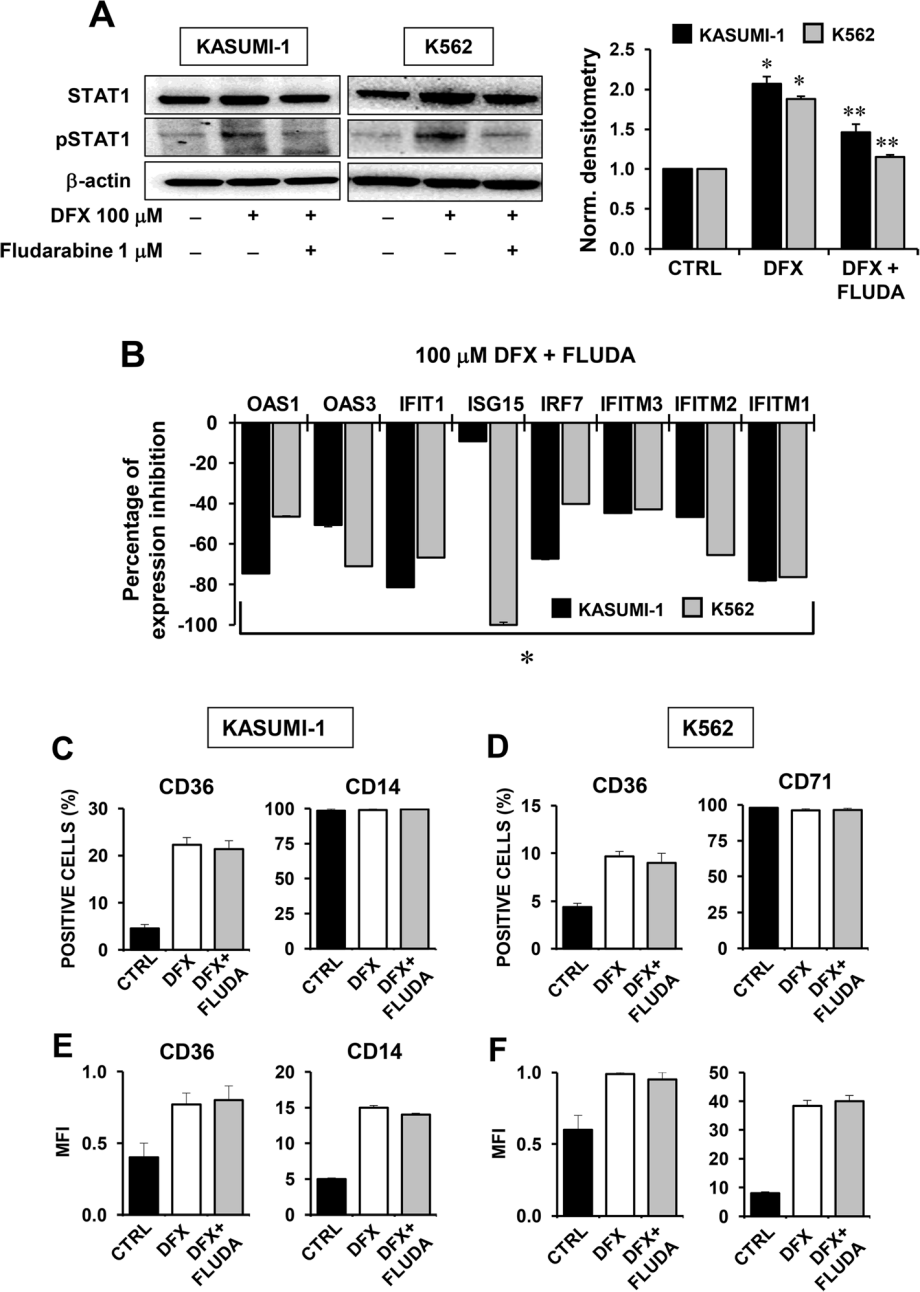
Effect of STAT1 inhibition on DFX-mediated differentiation in Kasumi-1 and K562 cells. **a** Western blot analysis of total STAT1 and pSTAT1^tyr701^ on whole protein Kasumi-1 (left) and K562 (right) cell extracts. Cells were treated with 100 μM DFX for 24 h ± a pre-incubation with 1 μM fludarabine (FLUDA) for 1 h. Per cell type, representative blots and bar histograms of densitometric analysis showing mean ± SEM p-STAT1/STAT1 ratio of three independent experiments are shown (*p < 0.05 vs CTRL; **p < 0.05 vs DFX). **b** Percentage of expression-inhibition of IFN-related gene transcript levels in Kasumi-1 and K562 cells treated with 100 μM DFX for 24 h + a pre-incubation with 1 μM fludarabine (FLUDA) for 1 h, compared to 100 μM DFX only treatment (*p < 0.05 vs DFX-only-treated cells). **c**, **e** Cytofluorimetric analysis of CD36 (left) and CD14 (right) expression in Kasumi-1 cells evaluated as percentage of positive cells (**c**) and as mean of fluorescence intensity (MFI) (**e**). Bars represent the mean ± SEM of three independent experiments (*p < 0.05 vs CTRL). **d**, **f** Cytofluorimetric analysis of CD36 (left) and CD71 (right) expression in K562 cells evaluated as percentage of positive cells (**d**) and as mean of fluorescence intensity (MFI) (**f**). Bars represent the mean ± SEM of three independent experiments (*p < 0.05 vs CTRL)

To disentangle the role of the IFN-related signalling and subsequent STAT1 activation on differentiation processes induced by DFX, we tested the STAT1-specific inhibitor fludarabine. Pre-treatment of the Kasumi-1 and K562 cell lines with 1 μM fludarabine for 1 h before DFX administration resulted in decreased STAT1 phosphorylation (Fig. 6a) as well as in inhibition of the DFX-mediated IFN-related gene expression (Fig. 6b). However, and notably, direct inhibition of STAT1 did not result in any effect on the DFX-mediated upregulation of the differentiation markers in both the leukemic cell lines as inferred from the flow-cytometric analysis shown in Fig. 6c–f. This last result strengthens the role of ROS signalling as major regulator of the differentiation process elicited by DFX.

## Discussion

Although iron chelation therapy with deferasirox proved to improve haematopoiesis in patients with bone marrow dysfunction, the mechanisms through which deferasirox exerts this action are currently unknown. In a previous study, we demonstrated the involvement of redox signalling in DFX-mediated effects on the self-renewal/differentiation process of healthy HS/PCs [16]. The in vitro differentiation assay performed with CD34^+^ cells from hUCB or hmBM here reported represents a functional validation of the ability of DFX to generate a greater number of BFU-E compared to control, thus confirming its aptitude to drive healthy stem cells towards a prominent erythroid differentiation. In particular, this effect was more evident in colonies from hUCB-derived CD34^+^ cells likely because of their prominent state of naïve cells [22]. Indeed, mobilization of adult HS/PC from bone marrow is known to induce a more rapid maturation towards committed progenitor cells [23, 24]. The slight but significant increase of BFU-E from adult HS/PCs was however detectable and accompanied by a strong reduction of CFU-GEMM/CFU-GM. Furthermore, NAC ability to counteract the observed DFX effects confirmed the involvement of ROS-mediated signalling in controlling the differential commitment program.

To deepen our investigations, we extended the analysis also to pathological models. To this aim, we chose three different leukemia cell lines: Kasumi-1, a leukemic myeloblastic cell line (FAB M2), characterized by a block of differentiation [25, 26]; K562, a cellular model of chronic myeloid leukemia, well known to study erythroid differentiation (FAB M1) [27] and the human promyelocytic HL60 cell line (FAB M2), able to differentiate to neutrophil-like cells in response to a variety of chemical stimuli [28]. We firstly tested the impact of DFX treatment on ROS production, confirming that DFX induced a significant increase of cellular peroxides and mitochondrial superoxide, suggesting mitochondria as a prominent source of ROS. Loss of the stemness phenotype and progression towards differentiation are accompanied with an increasing number of mitochondria and rewiring of cell metabolism towards oxidative phosphorylation [18]. Consistent with our previous finding in healthy HS/PCs, also confirmed by the gene expression analysis, DFX treatment elicited a significant increase of the mitochondrial mass and mtDNA copy number in Kasumi-1, K562 and HL60 cells. Collectively, these results support the involvement of the mitochondrial machinery and redox signalling in the regulation of cell fate, as already described [18, 29], and further corroborate the model whereby a slight ROS enhancement is able to orchestrate differentiation processes [30–32].

To test this assumption in the context of the observed effects of DFX, we evaluated whether DFX was able to drive differentiation processes through ROS activation in the leukemia cell models. The significant ROS-sensitive increase of BFU-E from DFX-treated K562 together with the induced expression of the erythroid differentiation markers CD36 and CD71, confirmed the DFX ability to drive erythropoiesis via redox signalling. The same analysis carried out with Kasumi-1 and HL60 cell lines led, however, to apparently contradictory results. Indeed, though DFX treatment induced NAC-sensitive upregulation of the differentiation markers of the myeloid lineage, this did not translate in an increase of the CFU-GM. A possible explanation might stem on the more mature differentiation stage of Kasumi-1 and HL60 (as compared to K562 (M2 versus M1)), which further boosts towards lineage commitment and maturation by DFX hampering their ability to form colonies as already described [33]. Unlike Kasumi-1 cells, ROS-dependent activity of DFX to form colonies was not confirmed in HL60 maybe because of their peculiar susceptibility to NAC exposure [34].

To identify any potential targets of DFX, we outlined a whole-gene expression profile of HS/PCs after DFX treatment. Ingenuity pathway analysis of the differential results attained indicated mitochondrial dysfunction and oxidative phosphorylation categories as those exhibiting the greatest statistically significant changes. However, IPA was unable to assign an unequivocal score in terms of activation or inhibition of the respective pathways. This is consistent with the observed altered redox homeostasis and enhanced biogenesis of the mitochondrial compartment in CD34^+^ HS/PCs following DFX treatment. In addition, and surprisingly, IPA pinpointed the IFN signalling as the main pathway activated by DFX. This effect appears specific since it is not observed with DFO, indicating differential iron-chelating properties. A growing amount of evidence indicates that IFN may exert a negative or positive regulatory effect on haematopoiesis in response to physiological or pathological conditions [35, 36]. A possible association between iron chelation and interferon signalling has been already described in cancer cells, both in haematological [37, 38] and in solid cancer models [39], suggesting that IFN pathway may be involved in anti-proliferative mechanisms during iron chelation. The strong increase in the expression levels of the IFN signalling genes (ISGs) observed in DFX-treated healthy HS/PCs was confirmed in the DFX-treated Kasumi-1 and K562 leukemia cell lines irrespective of their different maturation stage (M2 versus M1) or ability to generate different colonies (CFU-GM versus BFU-E). Most notably, the DFX-induced activation of the ISGs was NAC-sensitive both in healthy HS/PCs and in leukemic cells thereby suggesting a common redox signalling-mediated mechanism of action.

**Fig. 7:**
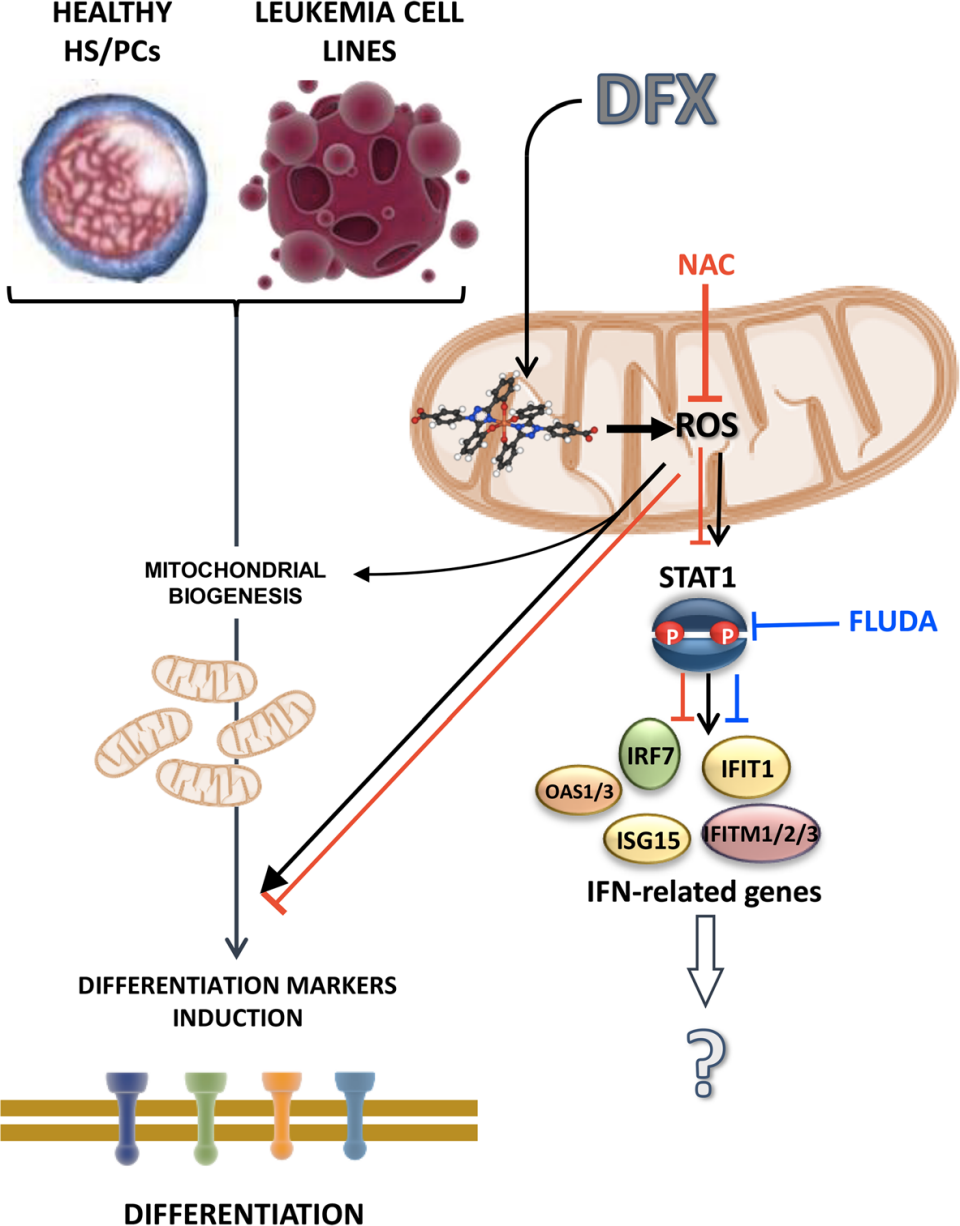
Proposed mechanism of action of DFX in healthy HS/PCs and leukemia cell lines. DFX is shown to induce enhanced mitochondrial ROS generation thereby activating redox signalling-mediated mitochondrial biogenesis and differentiation. In addition, ROS promote stabilization of the active phosphorylated state of STAT1, inducing the expression of IFN-related genes. NAC co-incubation (red line) counteracts DFX downstream effects while FLUDA treatment (blue line), although inhibiting STAT1 activation and IFN-related genes expression, is not able to affect DFX-induced differentiation. See “Discussion” for further details

In the attempt to deepen the DFX-responsive pathway(s), we focused on the transcription factor STAT1 whose activated phosphorylation state is well-known to be part of the IFN-related signalling and, in addition, to be sensitive to the cellular redox tone. Indeed, a number of studies showed that STAT1 activation/phosphorylation is induced, under stressing condition, by enhanced ROS production [40]. This would be achieved by activation of the redox-sensitive P38 MAPK and/or inhibition of redox-sensitive protein phosphatases [41]. Phosphorylation of STAT1 would promote its translocation as dimer into the nucleus and consequent binding to the interferon-stimulated response elements of the ISG promoters. The results attained clearly indicated that DFX induced a significant increase of phosphorylated STAT1 in the Kasumi-1 and K562 cell lines, which was prevented by co-treatment with the antioxidant NAC. However, when the STAT1 inhibitor fludarabine was co-incubated with DFX, under conditions hampering STAT1 phosphorylation and ISG expression, no change in the expression of the DFX-induced upregulated differentiation markers was observed. This insightful results would place modulation of redox signalling upstream of the DFX-induced gene expression rewiring, which would result on the one hand in upregulation of the mitochondrial biogenesis and differentiation and on the other hand in a STAT1-mediated activation of ISGs (Fig. 7). However, activated ISGs do not appear to be involved in the DFX-induced differentiation.

How DFX elicit ROS production remains to be mechanistically defined. Other studies reported that DFX treatment induces enhanced ROS production [42, 43]. This would appear counter-intuitive since iron load is a well-known ROS-generating condition [44]. Since other iron-chelating drugs, such as DFO, do not induce ROS generation [16], this would rule out the effect induced by DFX as dependent on its iron-chelating activity. However, it is also possible that given the higher affinity to iron and lipophilicity of DFX, its effect could be linked to selective sequestration of weakly bound iron from redox active oxidoreductases in intracellular compartments (Fig. 7). Notably, in the present study, we found a specific induction of superoxide anion in mitochondria. In a recent study, it was found that iron chelation impairs iron-sulfur (Fe-S) cluster biogenesis which in turn results in defective functioning of Fe-S clusters contained in the respiratory chain complexes [45].

## Conclusions

In conclusion, we demonstrated that DFX treatment is able to influence differentiation processes both in healthy haematopoietic CD34^+^ stem/progenitor cells and in leukemia cell models, thus suggesting a general mechanism of action. A limit of our study is the lack of validation in MDS or leukemia patients but the strong heterogeneity of samples did not allow to describe a common model. However, the understanding of the molecular mechanism underlying the haematological response by DFX would enable to predict a patient’s ability to respond to the drug, to extend the treatment to patients for whom iron chelation therapy is not recommended or to anticipate the start of treatment, regardless of the iron overload.

## Additional files

Additional file 1: **Figure S1.** Qualitative and quantitative analysis of ROS induction in leukemia cells by confocal microscopy. A: Representative laser scanning confocal microscopy imaging of intracellular and mitochondrial ROS production in living cells treated with 100 μM DFX ± 10 mM NAC, assessed by DCF (upper panels) and Mitosox (lower panels) respectively. Magnification of selected areas (indicated by the white frame) is shown at the bottom of each panel. The images are representative of three different preparations of Kasumi-1 yielding similar results. The histograms on the right show the quantitative analysis of the DCF or Mitosox-related fluorescence/cell; the values are means ± SEM of three independent experiments under each condition wherein the digitalized fluorescence images from at least five randomly selected optical fields (each containing about 10 cells) were analysed (*p < 0.05 vs CTRL, ^#^*p* < 0.05 vs DFX). B: Representative laser scanning confocal microscopy imaging of mitochondrial mass assessed by the specific Mitotracker-red probe in living Kasumi-1 cells treated with 100 μM DFX for 24 h. Magnification of selected areas (indicated by the white frame) is shown at the bottom of each panel. The histogram on the left shows the quantitative analysis of pixel intensity of probe-related fluorescence/cell; the values are means ± SEM, referring to at least ten optical fields randomly selected for each condition and clustered from three independent cell preparations (*p < 0.05 vs untreated cells (CTRL)). (TIF 10263 kb)

Additional file 2: **Figure S2.** DFX induces the expression of differentiation markers in leukemia cell lines. Representative flow cytometric analysis of differentiation markers in Kasumi-1, K562 and HL60 treated with DFX for 24 h [untreated (CTRL, black area) and DFX-treated (white area)]. CD36 and CD14 distribution was evaluated in Kasumi-1; CD36 and CD71 distribution was analysed in K562; CD11b and CD16 distribution was detected in HL-60. Per marker, histograms concerning percentage of positive cells (expressed as mean ± SEM) are shown (*p < 0.05 vs CTRL, ^#^*p* < 0.05 vs DFX). In cases where the percentage of positive cells was approximately 100% already at the basal level, we also reported the mean fluorescence intensity (MFI). (TIF 4972 kb)

Additional file 3: **Figure S3.** Differentially expressed genes associated with interferon signalling derived from IPA. Nodes representing gene products are displayed by cellular localization (extracellular space, plasma membrane, cytoplasm or nucleus). Genes in red are included in the dataset of differentially expressed genes in DFX-treated cells compared to control. (TIF 9688 kb)

Additional file 4: **Figure S4.** Ingenuity pathway analysis (IPA) of differentially expressed genes in CD34^+^ hBM-HS/PCs treated with DFO. Top 10 canonical pathways identified by IPA “Core Analysis” from genes changed more than 1.1-fold (*p* ≤ 0.05). Bars represent −log (*p* value) for significance; orange lines represent the ratio of changed genes to the total number of genes in the specific pathway. The IPA predicted one pathway having a positive *z*-score (predicted activation, red bar), one pathway having no activity/inhibition pattern predictable, in which *z*-score could not be calculated. The *z*-score of zero corresponded to the standard mean of the normal distribution curve. (TIF 5129 kb)

Additional file 5: **Table S1.** List of up- and downregulated genes in DFX-treated compared to DFX-untreated cells. (XLS 24 kb)

Additional file 6: **Table S2.** List of up- and downregulated genes in DFO-treated compared to DFX-untreated cells. (XLS 12 kb)

## Data Availability

All data generated or analysed during this study are included in this published article [and its supplementary information files].

## Abbreviations

BFU-E: Burst-forming unit-erythroid
CFU-GEMM: Colony-forming unit-granulocyte, erythroid, macrophage megakaryocytes
CFU-GM: Colony-forming unit-granulocyte/macrophage
DCF: Dichlorofluorescein
DFO: Deferoxamine
DFX: Deferasirox
GEA: Gene expression analysis
hBM: Human bone marrow
HS/PCs: Haematopoietic stem/progenitor cells
hUCB: Human umbilical cord blood
IFN: Interferon
IPA: Ingenuity Pathway Analysis
ISGs: Interferon signalling genes
MDS: Myelodysplastic syndrome
NAC: *N*-Acetyl cysteine
ROS: Reactive oxygen species
