# Supplementary figures and images for: Deferasirox drives ROS-mediated differentiation and induces interferon-stimulated gene expression in human healthy haematopoietic stem/progenitor cells and in leukemia cells
Source: Stem Cell Res Ther. 2019 Jun 13;10:171. doi: 10.1186/s13287-019-1293-y (PMC6567456; doi:10.1186/s13287-019-1293-y)

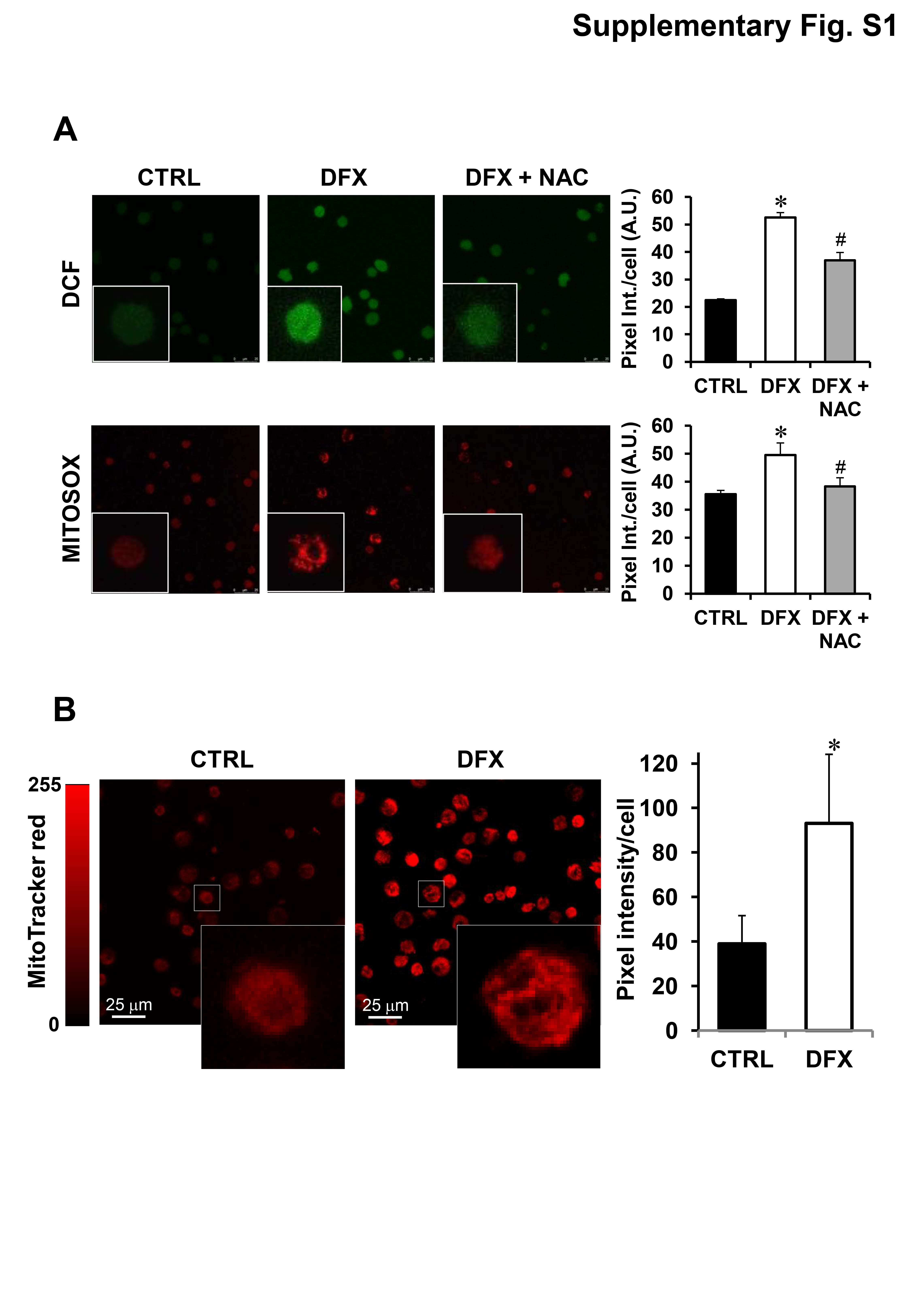

Supplement: Supplementary file 1 — Figure S1. Qualitative and quantitative analysis of ROS induction in leukemia cells by confocal microscopy. A: Representative laser scanning confocal microscopy imaging of intracellular and mitochondrial ROS production in living cells treated with 100 μM DFX ± 10 mM NAC, assessed by DCF (upper panels) and Mitosox (lower panels) respectively. Magnification of selected areas (indicated by the white frame) is shown at the bottom of each panel. The images are representative of three different preparations of Kasumi-1 yielding similar results. The histograms on the right show the quantitative analysis of the DCF or Mitosox-related fluorescence/cell; the values are means ± SEM of three independent experiments under each condition wherein the digitalized fluorescence images from at least five randomly selected optical fields (each containing about 10 cells) were analysed (*p < 0.05 vs CTRL, #p < 0.05 vs DFX). B: Representative laser scanning confocal microscopy imaging of mitochondrial mass assessed by the specific Mitotracker-red probe in living Kasumi-1 cells treated with 100 μM DFX for 24 h. Magnification of selected areas (indicated by the white frame) is shown at the bottom of each panel. The histogram on the left shows the quantitative analysis of pixel intensity of probe-related fluorescence/cell; the values are means ± SEM, referring to at least ten optical fields randomly selected for each condition and clustered from three independent cell preparations (*p < 0.05 vs untreated cells (CTRL)). (TIF 10263 kb) [file 13287_2019_1293_MOESM1_ESM.tif]

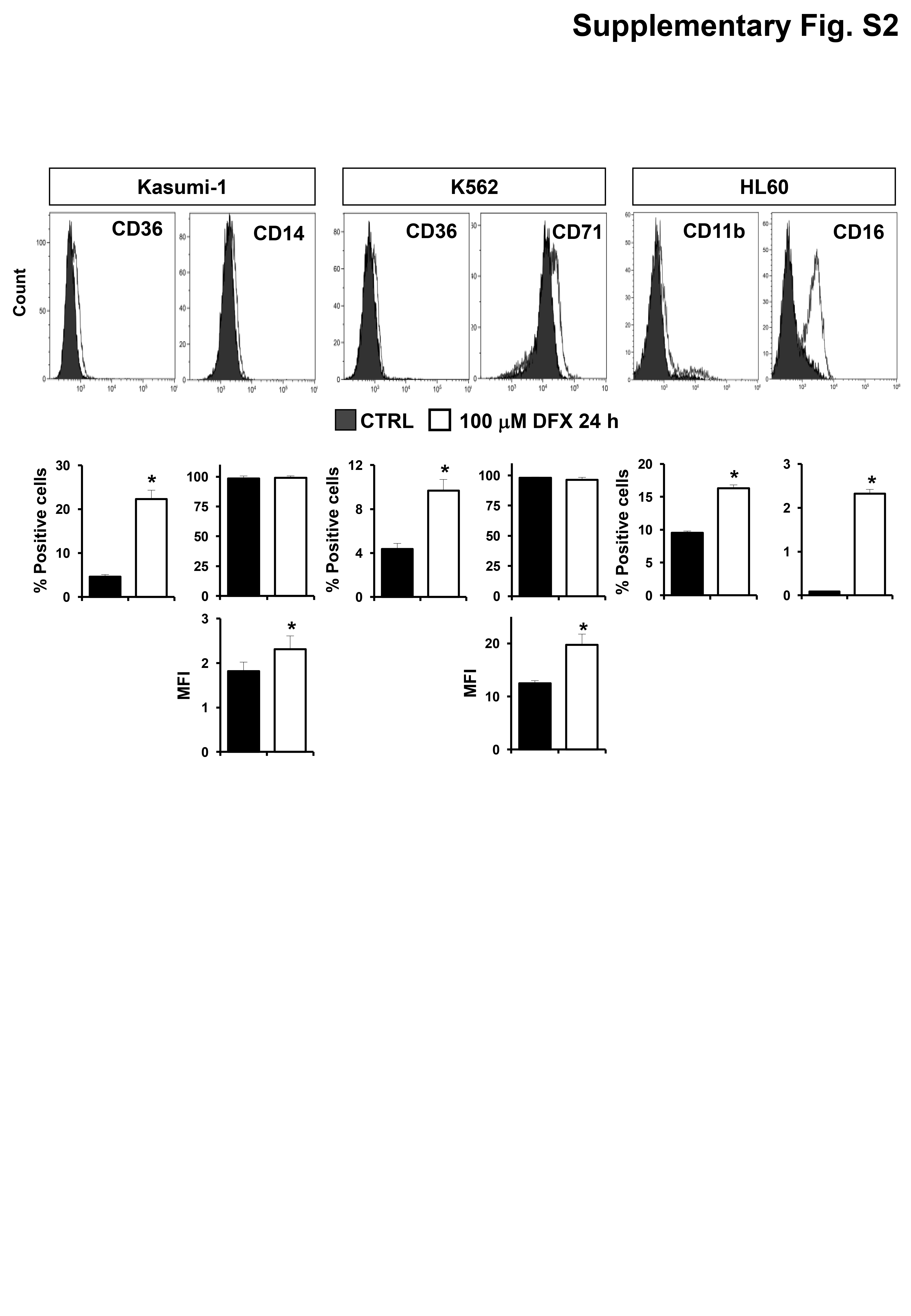

Supplement: Supplementary file 2 — Figure S2. DFX induces the expression of differentiation markers in leukemia cell lines. Representative flow cytometric analysis of differentiation markers in Kasumi-1, K562 and HL60 treated with DFX for 24 h [untreated (CTRL, black area) and DFX-treated (white area)]. CD36 and CD14 distribution was evaluated in Kasumi-1; CD36 and CD71 distribution was analysed in K562; CD11b and CD16 distribution was detected in HL-60. Per marker, histograms concerning percentage of positive cells (expressed as mean ± SEM) are shown (*p < 0.05 vs CTRL, #p < 0.05 vs DFX). In cases where the percentage of positive cells was approximately 100% already at the basal level, we also reported the mean fluorescence intensity (MFI). (TIF 4972 kb) [file 13287_2019_1293_MOESM2_ESM.tif]

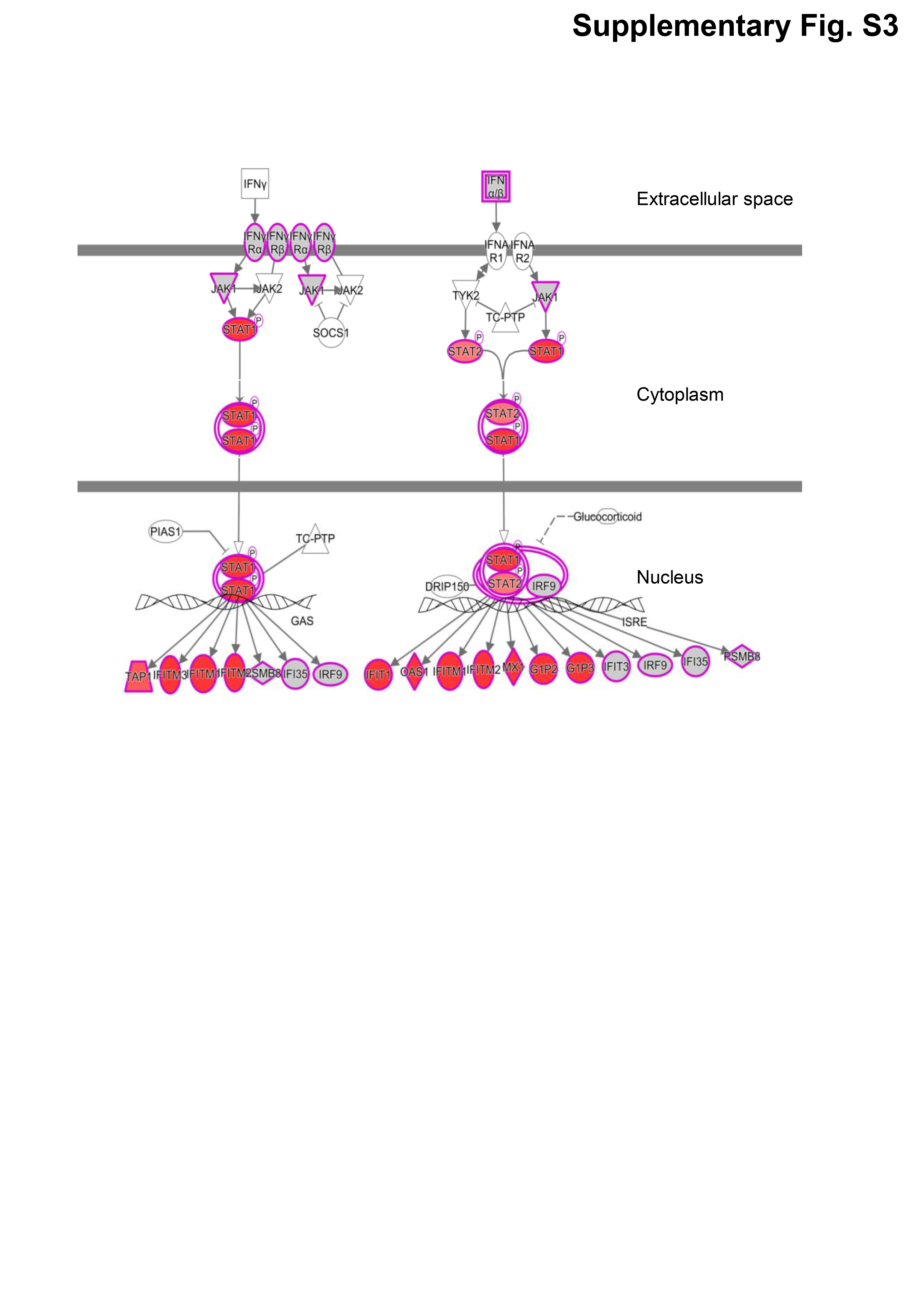

Supplement: Supplementary file 3 — Figure S3. Differentially expressed genes associated with interferon signalling derived from IPA. Nodes representing gene products are displayed by cellular localization (extracellular space, plasma membrane, cytoplasm or nucleus). Genes in red are included in the dataset of differentially expressed genes in DFX-treated cells compared to control. (TIF 9688 kb) [file 13287_2019_1293_MOESM3_ESM.tif]

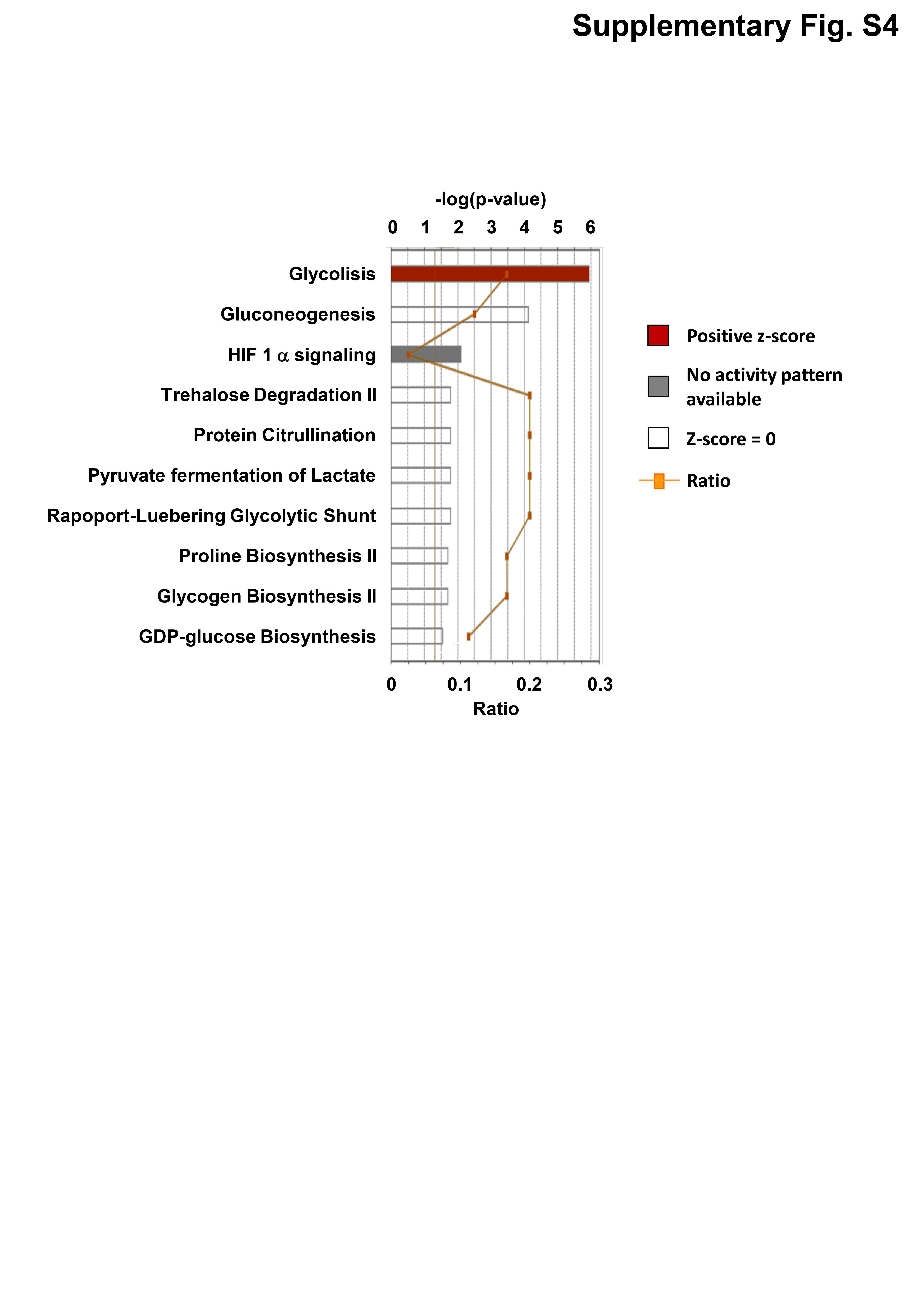

Supplement: Supplementary file 4 — Figure S4. Ingenuity pathway analysis (IPA) of differentially expressed genes in CD34+ hBM-HS/PCs treated with DFO. Top 10 canonical pathways identified by IPA “Core Analysis” from genes changed more than 1.1-fold (p ≤ 0.05). Bars represent −log (p value) for significance; orange lines represent the ratio of changed genes to the total number of genes in the specific pathway. The IPA predicted one pathway having a positive z-score (predicted activation, red bar), one pathway having no activity/inhibition pattern predictable, in which z-score could not be calculated. The z-score of zero corresponded to the standard mean of the normal distribution curve. (TIF 5129 kb) [file 13287_2019_1293_MOESM4_ESM.tif]
